# Comparability of *in Vitro* Tests for Bioactive Nanoparticles: A Common Assay to Detect Reactive Oxygen Species as an Example

**DOI:** 10.3390/ijms141224320

**Published:** 2013-12-13

**Authors:** Matthias Roesslein, Cordula Hirsch, Jean-Pierre Kaiser, Harald F. Krug, Peter Wick

**Affiliations:** 1 Empa, Materials-Biology Interactions Laboratory, Swiss Federal Laboratories for Materials Science and Technology, Lerchenfeldstrasse 5, CH-9014 St. Gallen, Switzerland; E-Mails: matthias.roesslein@empa.ch (M.R.); cordula.hirsch@empa.ch (C.H.); jean-pierre.kaiser@empa.ch (J.-P.K.); 2 Empa, Head of Research Focus Area Health & Performance, Swiss Federal Laboratories for Materials Science and Technology, Lerchenfeldstrasse 5, CH-9014 St. Gallen, Switzerland; E-Mail: harald.krug@empa.ch

**Keywords:** chemical reaction control, comparability, DCF assay, interference, nanoparticles, reactive oxygen species (ROS), Sin-1

## Abstract

The release of reactive oxygen species (ROS) during the electron transport of mitochondrial aerobic respiration is the major source of ROS. However, contact between cells and nanoparticles (NPs) can also induce release of ROS, leading to an imbalance towards the pro-oxidative state. At low levels of ROS production, cells initiate a protective response to guarantee their survival, but an excess of ROS can damage cellular compounds such as membranes and various organelles, or directly cause genotoxicity. Thus an elevated level of ROS is an important indicator of cellular stress and an accurate recording of this parameter would be very informative. ROS can be measured by various assays, but all known assays measuring and quantifying ROS possess certain weaknesses. The problems and challenges of quantitatively detecting ROS *in vitro* using the 2′,7′-dichlorodihydrofluorescein (DCF) assay is discussed as an example. In addition, we debate the difficulties in finding a suitable and stable chemical reaction control for the DCF assay (or other ROS-detecting assays). As a conclusion, we believe that using 3-morpholinosydnonimine hydrochloride (Sin-1) as a ROS inducer in the DCF assay is feasible only qualitatively. However, a quantitative measurement of the absolute amount of ROS produced and a quantitative comparison between experiments is (at the moment) impossible.

## 1. Introduction

Nanoparticles (NPs) are materials with three external dimensions in the nanoscale range (1–100 nm). The characteristics of NPs are their small size, large surface area, and often completely different material properties compared to their micro-sized counterparts. Their large surface area confers the novel mechanical, electrical, thermal and optical properties of NPs [1]. Because of the widespread use of NPs in consumer products, technical applications, medical treatments, *etc.*, industrial production has increased tremendously in the past decade and public exposure to engineered NPs may increase in parallel.

A decrease in particle size can lead to an increase in the number of structural defects, as well as disruption of the well-structured electronic configuration of a material, which could establish specific surface groups that function as reactive sites [2]. Thus, the toxicity of NPs is at least partly related to the surface reactivity of the particle, which may also be relevant to health effects [2–5]. In addition, many of the NPs that are synthesized today are metallic, such as nanosilver or nanocopper, which under certain conditions release metal ions. The chemical properties of metal ions enable their uptake via the cell membrane’s ion transporters [6,7]. It has been demonstrated that metal salts and metal ions, such as certain NPs with reactive sites on their surfaces, can induce oxidative stress through generation of reactive oxygen species (ROS) [8–15].

ROS is a collective term for different reactive molecules and free radicals derived from molecular oxygen. The most common ROS include superoxide anion (O_2_^−^), hydrogen peroxide (H_2_O_2_), hydroxyl radical (HO^•^) and singlet oxygen (^1^O_2_), all of which are more reactive than oxygen (O_2_) itself. The molecules are produced during the electron transport of mitochondrial aerobic respiration or by oxidoreductase enzymes and metal catalyzed oxidation. ROS are not only involved in the induction of cell death, but also regulate the expression of certain genes and are able to activate cell signaling cascades [16].

**Figure 1.**
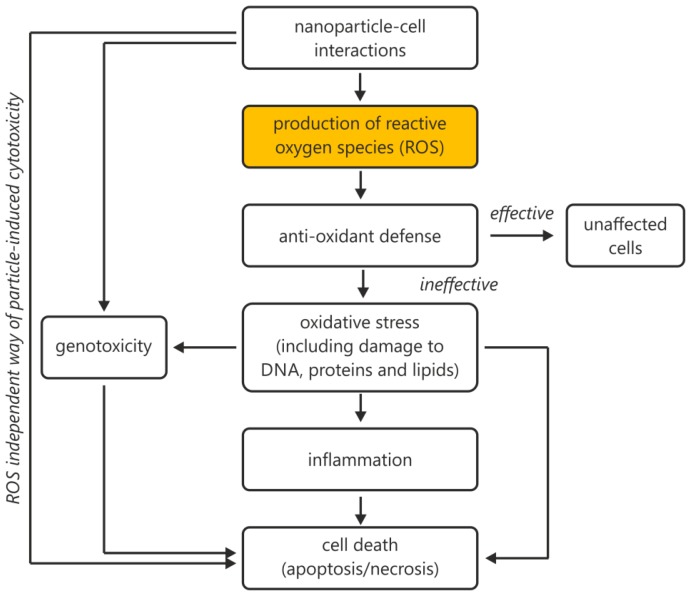
Nanoparticle–cell interactions with a central role for reactive oxygen species (ROS). Summary of possible cellular reactions taking place after nanoparticles (NPs) interact with cellular systems.

Another method of ROS generation involves NPs and their interaction with cells (either simple contact or internalization of NPs into cells). The catalytically active surface of the NP can generate an increased level of ROS as a fast response (Figure 1).

At low levels of ROS production, cells initiate a protective response to promote their survival. This can induce transcription factors, such as Nrf2, which increase the antioxidant defense of the cells, leading to removal of excess ROS and thereby to detoxification. ROS generated at low frequency are easily neutralized by antioxidant defenses, such as glutathione (GSH) and antioxidant enzymes, which balance the production and destruction of ROS. An imbalance toward the pro-oxidative state is often referred to as oxidative stress [17].

Under conditions of excess ROS production, the natural antioxidant defenses may be overwhelmed and at higher levels of ROS the protective response is overtaken by inflammation and cytotoxicity. Inflammation is initiated through the activation of pro-inflammatory signaling cascades, such as mitogen-activated protein kinase (MAPK) and nuclear factor κB (NFκB) [2,18]. An excess of ROS can cause apoptosis. NFκB inhibits apoptosis by up-regulating several anti-apoptotic genes [19].

Activated transcription factor NFκB stimulates the production of inflammatory cytokines (e.g., tumor necrosis factor alpha (TNF-α), interleukin (IL-x)). The release of cytokines has the potential to cause inflammation under *in vivo* conditions and can ultimately promote the development of diseases (fibrosis, cancer, apoptosis, and necrosis) [20]. The formation of free radicals at the surface of NPs or by metallic ions is according to Donaldson *et al.* responsible for damaging supercoiled DNA [20]. Thus an increased level of ROS can be directly genotoxic. Furthermore, it has been reported that higher levels of released ROS lead to cellular constituents, such as membranes, proteins, lipids *etc.*, being attacked.

ROS are produced by all cell types and serve as important messengers for both intra- and intercellular communication [21]. With respect to intracellular signaling, H_2_O_2_ is the most interesting candidate, whereas nitric oxide (NO^•^) is involved primarily in intercellular signaling [22]. ROS molecules are highly reactive and interact with various molecules. This lack of specificity makes the search for a specific biomarker of a discrete ROS molecule nearly impossible [22]. Nowadays, ROS are measured by various assays, but unfortunately all known tests have their limitations. The most popular tests for estimating the level of ROS in *in vitro* systems are colorimetric, fluorescent or chemiluminescent dyes (e.g., 2′,7′-dichlorodihydrofluorescein-diacetate (H_2_DCF-DA) assay or NO^•^ assay), as well as methods using electron spin resonance (ESR), and assays such as glutathione peroxidase or lipid peroxidation.

Our aim is to provide a short overview of the most frequently used ROS assays. A further goal is drawing attention to the difficulty of finding an appropriate and stable chemical reaction control for ROS-detecting assays. We discuss as an example the use of 3-morpholinosydnonimine (Sin-1) as a ROS inducer in the DCF assay. The use of Sin-1 is a catch-22 situation and as a consequence it is still impossible to quantitatively measure the amount of released ROS.

### 1.1. Brief Description of the Different Assays and Their Weakness

#### 1.1.1. Fluorescent and Chemiluminescent Assays

The measuring of HO^•^ is generally performed with fluorogenic or chemiluminogenic substances, which serve as hydrogen donors. The commonly used substrates for these types of assays are H_2_DCF-DA [19], homovanillic acid [23], and AmplexRed [24]. Unfortunately, a number of these fluorogenic substrates can only efficiently detect the cellular peroxides in the presence of a peroxidase (e.g., horseradish peroxidase, HRP) or a transition metal [25,26]. The HRP or transition metal decomposes the peroxides to radicals, which are able to react with the dye. However, the use of HRP in the assay can lead to incorrect results, because cellular compounds, such as thiols can serve as a substrate for HRP and thus affect the fluorescence by either enhancing or quenching the signal [22].

The oxidation of H_2_DCF has been used quite extensively for the quantitation of different types of ROS [26–29]. H_2_DCF is applied as H_2_DCF-DA, which is taken up by the cell, where unspecific esterases cleave the lipophilic groups, resulting in a charged compound. Oxidation of H_2_DCF by ROS converts the molecule to DCF, which is fluorescent. It is important that the test is done in a medium free of serum (e.g., Hank’s buffered salt solution; HBSS), because serum contains endogenous esterases, which will cleave the ester group of H_2_DCF-DA. The active dye in the medium will react with oxidizing compounds, which will influence the measurement result [26]. Deacetylated H_2_DCF is sometimes retained intracellulary [28,30] allowing measurement of specifically intracellular ROS production. However, some leakage of the dye is possible [26], leading to non-ionic H_2_DCF molecules accumulating in the medium, where it is free to interact with oxidants and can undergo extracellular reactions. H_2_DCF, like many other fluorescent substrates, is not highly specific, which can be an advantage in assays where different types of ROS are produced or in assays where a certain nanocompound induces the release of unknown ROS. Furthermore, DCF has a high fluorescence quantum yield and hence is detectable by fluorescence spectroscopy.

#### 1.1.2. Nitric Oxide Assay

Dihydrorhodamine (DHR) is another frequently used substrate for measuring reactive molecules and free radicals, such as nitric oxide (NO^•^), peroxynitrate (ONOO^−^) or hydroxyl radicals (HO^−^). In this assay, the reduced dye, DHR, is oxidized by ROS, respectively by free radical oxidants derived from them, to its corresponding fluorescent product, rhodamine [31]. This test also has its limitations. The oxidative conversion of DHR to rhodamine is mediated by an intermediate DHR radical, which is usually further oxidized by oxygen to yield a stable fluorescent product. The reaction of the intermediate DHR radical can cause an artificial enhancement of the fluorescent signal intensity [28]. Further, the DHR conversion is not very specific because DHR reacts with NO^•^, as well as with superoxide and several other oxidants.

#### 1.1.3. Electron Spin Resonance Assay

ESR, also known as the electron paramagnetic resonance technique, is the only technique that allows the measurement of free radicals, such as HO^•^ and superoxide (O_2_^−^). Unfortunately, only fairly unreactive radicals can be detected with this technique. Reactive radicals do not accumulate to levels that are sufficiently high to allow detection [26]. Highly reactive radicals can be converted to relatively stable radicals by the spin-trapping technique [32]. A five-membered-ring nitron derivative, 5,5-dimethyl-1-pyrroline-*N*-oxide (DMPO), is widely considered to be the most powerful spin-trapping reagent [33]. ESR can then readily detect the more stable species. DMPO is commonly used for *in vitro* studies of living cells, but DMPO is cytotoxic at high concentrations, which is limiting its use [33]. Another limitation of the spin-trapping method is that a free radial can react immediately with a molecule other than the spin-trapping agent. Thus the spin-trapping adduct will not be generated and hence it is not detectable by ESR. A further drawback of the ESR method is reacting of the generated spin adduct with a reducing agent (if present) and its potential neutralization [33]. The relatively high cost of the equipment is another downside of this method [34].

#### 1.1.4. Glutathione Peroxidase Assay

Glutathione (GSH) is a three-amino-acid peptide, active in a non-enzymatic oxidant defense mechanism, which serves to detoxify peroxides and to regenerate a number of important antioxidants [22]. Different colorimetric and fluorescent-based assays are available to measure the amount of GSH/GSSG in *in vitro* cultures. GSH is regenerated from its oxidized form (GSSG) by the action of a NADPH-dependent reductase [22], which can influence the measurement results and the GSSG concentration might be underestimated.

#### 1.1.5. Lipid Peroxidation Assay

Lipid peroxidation is a ROS-mediated process by which polyunsaturated fatty acids undergo peroxidation, producing α, β-unsaturated aldehydes such as malondialdehyde (MDA) [35]. In contrast to other radical scavenging assays, the lipid peroxidation assay can evaluate test compounds in the presence of other antioxidants, iron and radicals [34]. Lipid peroxidation is one of the most widely used indicators of free radical formation. The previously described assays (H_2_DCF, NO, and ESR) directly measure the free radicals, whereas the lipid peroxidation assay does not measure released radicals, rather it measures the amount of oxidized fatty acids, which correlates with the amount of released radicals. Measurement of lipid peroxidation is generally done by detecting MDA generated from the decomposition of lipid peroxidation products. The big problem is that this method is not necessarily specific to MDA [36]. Various compounds have a similar absorption maximum as MDA, which leads to an overestimation of the MDA values [37]. Progress in mass spectrometry (MS) has prompted the development of more accurate and sensitive methods for various lipid peroxidation products [32]. One considerable advantage of MS-based methods is the ability to identify individual lipid species targeted by ROS and to detect the various oxidative products formed [32,38].

### 1.2. General Weakness in the Measurement and Quantification of ROS

All assays for measuring ROS possess a certain weakness, as discussed. In addition, all the assays are prone to numerous artifacts resulting from sample preparation or from the analytical method itself. Organic solvents, such as dimethyl sulfoxide (DMSO) or ethanol, which are used to dissolve the test compounds, are another difficulty that all tests have in common. DMSO and ethanol are powerful hydroxyl scavengers [34], which could lead to an underestimation of the amount of ROS produced. In addition, it is essential to evaluate the test compounds. Appropriate control samples are very important, because the results can be altered by various reactions taking place in the culture medium or by cell culture processes themselves that may lead to oxidative stress [21,26]. In several studies using H_2_DCF-DA to detect ROS, 3-morpholinosydnonimine hydrochloride (Sin-1) was used as the chemical reaction control [39–44]. Unfortunately, Sin-1 (as with any other ROS-inducing compound used as a chemical reaction control) is chemically unstable and so the amount of oxidized H_2_DCF varies considerably. As a consequence, any chemically unstable positive control will cause greatly varying intensities of the fluorescent signals in different independent experiments. It is this lack of a reliable fluorescent signal, obtained from a chemical reaction control, that prevents quantification of the effects (*i.e.*, ROS production) of a given unknown compound (*i.e.*, NP).

## 2. Results and Discussion

Four basic concepts [45] (*i.e.*, validation, quality management system, as outlined in ISO 17025 (2005) (Ref ISO17025(2005)) in detail, measurement uncertainty [46,47] and traceability [48]) all have to be thoroughly implemented and functioning for each assay method to achieve reliable and comparable results. If one of the concepts is not functioning, then comparability of results between and within laboratories is impossible. In the case of assays detecting ROS, it is traceability that fails, as we describe below for the DCF assay as an example.

### 2.1. Traceability as One of the Four Factors in Achieving Comparability

Outside the field of measurement science, the concept of traceability is often not well understood, despite being a precondition for comparing results within and between laboratories. We elaborate this concept for the DCF assay investigating the bioactivity of NPs and also highlight the serious difficulty of establishing it by comparing 24 individual experiments (see Section 2.1.2.). We also enumerate some aspects of the different chemical reaction controls that can reliably detect potential interference between NPs and the assay itself.

#### 2.1.1. Establishing Traceability for the DCF Assay: Theoretical Considerations

The principle of traceability demands, as a precondition, a clear and unambiguous definition of the measurand, which is the “quantity subjected to measurement” [47]. In the case of the DCF assay, the quantity of interest is the measured intensity of fluorescence from the DCF dye that has been oxidized by ROS in the cells. However, the system detects the total fluorescent signal of all generated DCF dye and because further reactions with other compounds in the assay system can also oxidize H_2_DCF, additional elaborate chemical reaction controls have to exclude any such interference.

**Figure 2.**
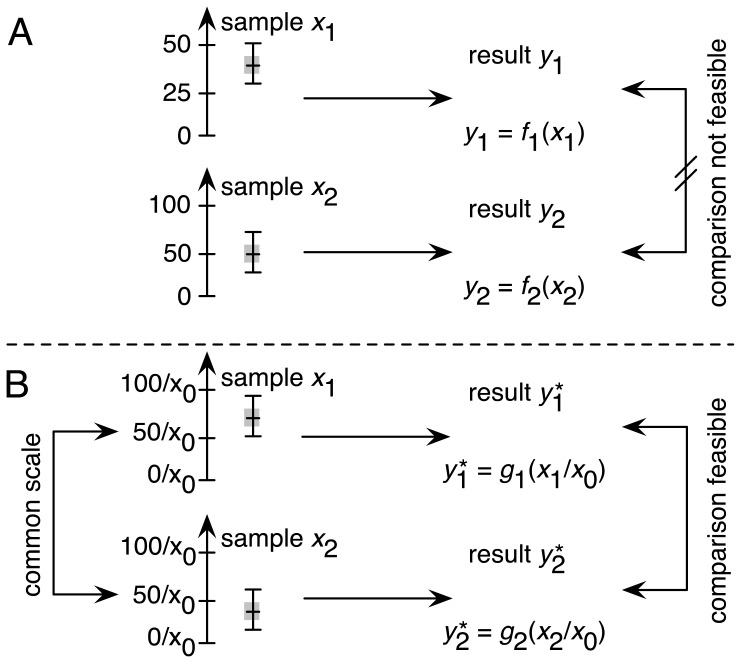
(**A**) Results *y*_1_ and *y*_2_ of the test sample *x*_1_ and of the test sample *x*_2_ cannot be compared, because no internal measurement scale establishes traceability; (**B**) The chemical reaction control sample *x*_0_ introduces an internal reference scale that enables the comparison of result $y_{1}^{*}$ with $y_{2}^{*}$. (*f*_1_, *f*_2_, *g*_1_ and *g*_1_ are the common symbols of a mathematical formula. In this case, they represent the equation of the measurement that relates to the test sample *x*_1_ with *x*_2_. Besides this, they relate to all other major effects in a mathematical form (model) with the measurand, which is the quantity to be measured).

Actual traceability is established by introducing a measurement scale that allows comparison of results [48]. Currently, it is not feasible to directly measure the amount of NPs interacting with a cell, so an indirect measurement scale uses the amount of NPs applied to the assay as a practicable alternative. This considerably affects the minimal set of parameters characterizing the NPs. Besides direct physical measures, such as chemical composition, diameter, shape and morphology *etc.*, indirect physical chemical properties, such as the degree of agglomeration/aggregation under experimental conditions, protein coating and surface charge, have to be known before the measurement scale for the NPs can be described with enough validity. All these parameters define a measurement scale describing the amount of “available” NPs that can interact with cells. Furthermore, our current knowledge of the interactions between NPs and cells is limited and therefore, prediction of the size of a bioactive reaction is not feasible. Even a small change in one of the many parameters describing NPs can cause considerable effects in cells (Figure 2A). The ever present batch-to-batch variability in NP production prevents creation of a stockpile of homogeneous NPs. Therefore an alternative approach has to be found to establish a measurement scale within the assay.

Figure 2B shows an alternative solution. The chemical reaction control is used not just as a 100% effect control, but also as a calibrator in a concentration series. This allows rigorous assessment of the assay’s performance by determining the EC_50_ in each measurement round. With that, we establish traceability between different measurements over time and space, because EC_50_ results can be compared for the chemical reaction controls (*x*_0_ in Figure 2B) of each experiment. If the EC_50_ results are in agreement, then we know that the assay is fully functional and so comparison of the results for NPs is feasible. Otherwise, if there are significant differences in the EC_50_ results of the chemical reaction controls, we know that there are some sources of errors or even blunders in the different experiments, which have to be investigated and detected before any further comparison of results can take place.

When a concentration series is prepared from the positive control, its chemical properties need to satisfy more stringent requirements. The chemical compound used should be of known purity and composition, chemically stable, easily weighable and readily available. Such a chemical compound is cadmium sulfate (CdSO_4_), which is often used for acute toxicity assays such as 3-(4,5-dimethylthiazol-2yl)-2,5-diphenyltetrazolium bromide (MTT) or 3-(4,5-dimethylthiazol-2-yl)-5-(3-carboxymethoxy phenyl)-2-(4-sulfophenyl)-2*H*-tetrazolium inner salt (MTS). CdSO_4_ is a white and easy weighable powder of known high purity, and can be dried if necessary. As the cadmium ion causes the acute toxic reaction, it is also inherently chemically stable.

Finding a suitable and chemically stable positive control for the DCF assay (or other ROS-detecting assays) has proven very difficult. ROS-inducing compounds are very short-lived species because of their radical nature. As such, their chemical precursors that are used practically as chemical reaction controls (e.g., H_2_O_2_ or Sin-1) must also exhibit considerable reactivity to produce the desired radical species (NO^•^, NO_3_^−^*etc.*). As an example, Sin-1 releases NO to spontaneously form peroxynitrite (ONOO^−^) when it is in solution [27]. Because the preparation of a dilution series takes time, a certain amount of Sin-1 will spontaneously decompose and will no longer be available for the induction of ROS in the cellular system. Accordingly, the actual concentration of the chemical reaction control is unknown after it has been added to the *in vitro* assay. Even for freshly prepared stock solution, the concentration will vary with the time taken to prepare the solution and then dilute it to the required concentrations.

This aspect causes great uncertainty about the concentration series of the chemical reaction control and hence a reliable and reproducible EC_50_ value cannot be determined. Lacking traceability, no direct quantitative comparison of any result is feasible. The extent of the concentration uncertainty also determines the usability of the DCF assay even as a qualitative measure of ROS activity as “severe”, “medium” or “light”. The lack of any chemically stable compound that can be used to generate any functional concentration series demands an alternative solution. On a qualitative level, at least, several readily available and suitable NPs that induce a range of ROS reactions in the DCF assay could be used to create a semi-quantitative ladder from “light” to “medium” to “severe” effects. Thus, the level of ROS production by an unknown NP could be gauged as lying between the “light” and “medium” reactions caused by two well-characterized NPs. Such NPs need to fulfill the criteria of a reference material and need to be available on a larger scale, because with each DCF assay part of this stock will be used up.

Beside this, any interference by the investigated NP with the DCF chromophore will further hinder the traceability chain by quenching the fluorescent signal. This demands a correction for the loss of signal in the equation of the measurand. Moreover such a quenching behavior does often not display a linear relation with the concentration of the NPs. Hence the overall uncertainty of the assay measurement is increased additionally disabling any quantitative statement. Also the proposed semi quantitative ladder needs to be tested for the effect of the interference, as soon as this ladder becomes available.

#### 2.1.2. Establishing Traceability for the DCF Assay: Practical Experiments Using Sin-1 as a Potential Chemical Reaction Control

**Figure 3.**
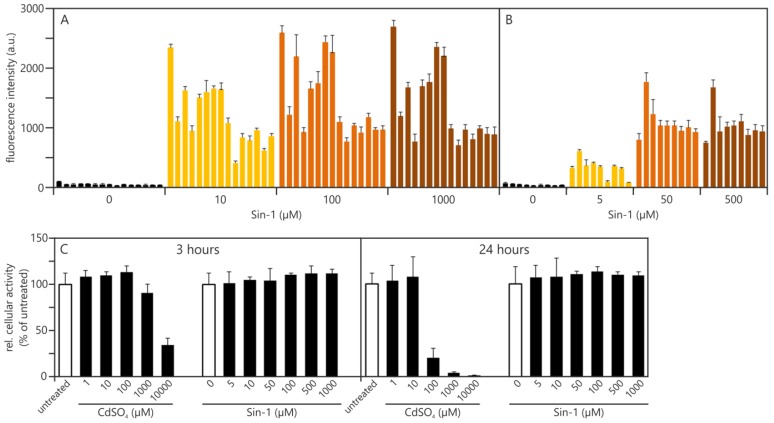
Absolute values for detecting reactive oxygen species (ROS): After one hour of H_2_DCF-DA loading, A549 cells were treated for three hours with different concentrations of Sin-1. Arbitrary fluorescence values after blank subtraction are shown. Each bar represents the mean of three technical replicates of an independent experiment and corresponding standard deviations. (**A**) 15 independent experiments using 10, 100 and 1000 μM Sin-1; (**B**) Nine additional independent experiments using reduced Sin-1 concentrations (5, 50, 500 μM); (**C**) Sin-1 concentrations used show no sign of toxicity in A549 cells. Cells were treated for three and 24 hours with the indicated Sin-1 concentrations. CdSO_4_ served as the chemical reaction control at both time points and induced dose- and time-dependent cytotoxicity.

**Figure 4.**
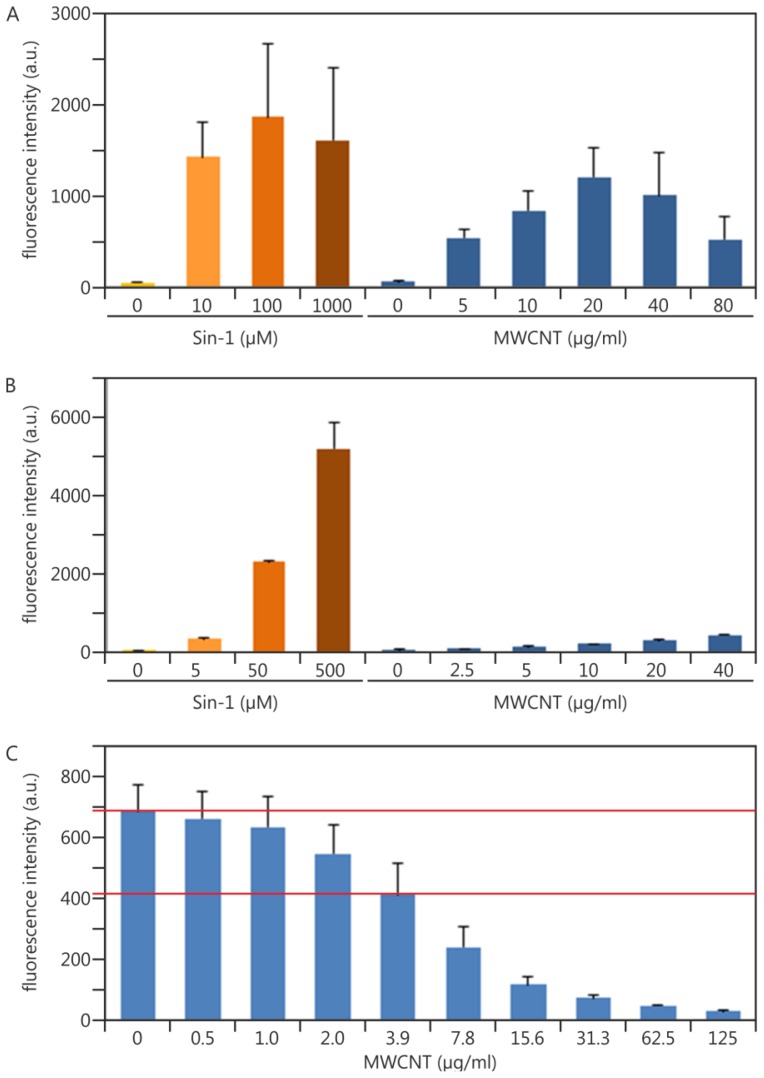
Cell-based 2′,7′-dichlorodihydrofluorescein (DCF) assay and corresponding cell-free nanoparticles (NP) interference controls: For each graph, three independent experiments and their standard deviations are shown. In each case, fluorescence was measured after three hours of incubation. (**A**) A549 cells treated with increasing concentrations of Sin-1 and multiwalled carbon nanotubes (MWCNTs), respectively; (**B**) The deacetylated form of H_2_DCF, incubated with increasing concentrations of Sin-1 and MWCNTs, in a cell-free environment to estimate the intrinsic oxidative potential of MWCNTs; (**C**) The already fluorescent molecule, DCF, incubated with increasing concentrations of MWCNTs to assess possible quenching effects.

Our initial experiments were carried out using a shortened dilution series of Sin-1 (10 μM, 100 μM, 1000 μM) (Figures 3A and 4A). Figure 3A shows 15 independent experiments performed over a time frame of almost two years. At a concentration of 10 μM Sin-1, a considerable amount of ROS can already be detected in every single experiment. However, relative fluorescence values varied between approximately 360 arbitrary units (a.u.) (Experiment 10) and 2200 a.u. (Experiment 1). Besides that, dose dependency is only obvious from 10 to 100 μM Sin-1 and only in some experiments (e.g., Experiment 10), while in other cases (e.g., Experiment 4) the maximum values are already reached at 10 μM of Sin-1. We investigated Sin-1 cytotoxicity using the MTS (3-(4,5-dimethyl-2-yl)- 5-(3-carboxymethoxyphenyl)-2-(3-sulfophenyl)-2*H*-tetrazolium) cell viability assay (Figure 3C). Even though microscopic inspection revealed morphological changes of cultures treated with 1000 μM Sin-1 no cytotoxicity could be detected at this concentration; not even after 24 h of incubation (Figure 3C). To match Sin-1 concentrations of cell-free (see Figure 4B and Experimental Section) and cellular experiments we reduced the Sin-1 concentrations in subsequent experiments to 5 μM, 50 μM and 500 μM. This reduction also allowed us to elucidate whether or not the observed morphological changes account for the reduced fluorescence values in 1000 μM Sin-1 treated samples. However, there was still no further increase in fluorescence intensity measureable between 50 and 500 μM. Values measured after treatment with 5 μM Sin-1 varied between 40 and 550 a.u. Thus, reducing the Sin-1 concentration offered no improvement in terms of quantitative traceability.

Nevertheless, because in each and every experiment Sin-1 treatment resulted in detectable induction of ROS we conclude that its usage as a ROS inducer in the DCF assay per se is possible. However, a quantitative estimation of the amount of ROS produced and its relation to an absolute scale is not possible. Hence quantitative comparison of one experiment to another is impossible and with it the establishment of traceability is not feasible.

#### 2.1.3. Experimental Data Adding Quality Control Measures in terms of NP Interference: Multiwalled Carbon Nanotubes as an Example

In parallel to the Sin-1 samples shown in Figure 3, we also performed NP treatments. Figure 4A shows induction of ROS by multiwalled carbon nanotubes (MWCNT) as an illustrative example. Sin-1 controls were performed as described and the results showed a highly similar profile to that shown in Figure 3. Multiwalled carbon nanotubes (MWCNTs) induced ROS in a dose-dependent manner up to 20 μg/mL. However, fluorescence decreased at higher concentrations. Microscopic observation as well as additional acute toxicity assays revealed no obvious signs of cell death (data not shown). Cell-free control experiments were performed to investigate two possibilities: (i) the NPs’ intrinsic ability to induce ROS (*i.e.*, to process the deacetylated H_2_DCF molecule without any involvement of cellular reactions); and (ii) a quenching effect caused by MWCNT agglomerates that reduced the existing fluorescent signal from the processed DCF dye.

Sin-1 was also able to process H_2_DCF in a cell-free environment and, as such, proved to be useful as a chemical reaction control in this setting. Nevertheless, and according to the cellular setup, this is only true in a qualitative manner because the absolute fluorescence values varied similarly (data not shown). As no cells were involved and hence cell death could not limit Sin-1 activity, we observe a dose-response relationship. MWCNTs themselves only marginally process the H_2_DCF molecule (Figure 4B).

Adding MWCNTs to the fluorescent DCF dye revealed a dose-dependent quenching effect of these NPs (Figure 4C). At a concentration of 3.9 μg/mL, a 40% reduction in fluorescence intensity was already observed. We conclude that the reduction in signal intensity detected in the cellular assay (Figure 4A) was due to this quenching effect. However, the quenching starts already at very low concentrations. It is thus most likely that the MWCNT preparation used here [46] induced even more ROS production in cells than can be detected with the DCF assay performed as described. It is impossible to re-calculate, extrapolate or even estimate the real level of ROS production in cells from our cell-free controls. Rather, the control results served as qualitative evidence for whether or not interference by a particular type of NP occurred.

## 3. Experimental Section

### 3.1. Nanoparticles

We used MWCNTs from Bayer Technologies Service (Baytubes^®^, Leverkusen, Germany) and a thorough characterization has already been published [46]. For our experiments, MWCNTs were dispersed at a stock concentration of 250 μg/mL by sonication (10 min ultrasonic bath) in ultrapure water containing 160 ppm Pluronic F127 (Sigma, Buchs, Switzerland). Because MWCNTs are hydrophobic, the non-ionic biocompatible detergent Pluronic F127 was used to obtain homogeneous suspensions.

### 3.2. Cell Culture

The human alveolar epithelial cell line A549 (ATCC: CCL-185) [48] was obtained from American Type Culture Collection and grown in RPMI-1640 cell culture medium (Sigma, Buchs, Switzerland) supplemented with 10% fetal calf serum (FCS, Lonza, Visp, Switzerland), 0.2 mg/mL l-glutamine (Gibco, Life Technologies, Zug, Switzerland), 50 μg/mL penicillin (Gibco, Life Technologies, Zug, Switzerland), 50 μg/mL streptomycin (Gibco, Life Technologies, Zug, Switzerland) and 100 μg/mL neomycin (Gibco, Life Technologies, Zug, Switzerland) in humified air at 37 °C and 5% CO_2_. Cells were subcultured at approximately 80%–90% confluency using 0.5% Trypsin-EDTA (Sigma, Buchs, Switzerland).

### 3.3. Determination of ROS by DCF Assay

#### 3.3.1. Preparation and Handling of Sin-1

A 1 mM stock solution of Sin-1 (Sigma, Buchs, Switzerland; #M5793) is prepared in HBSS and either immediately further diluted in HBSS or frozen in single-use aliquots. This step has to be performed as fast as possible. Dilutions are prepared as close to the time of usage as possible. Any delay between preparation of stock (or thawing of the stock aliquot), dilution and cell treatment has to be avoided. Because of the reactive and thus unstable nature of Sin-1, any delays and handling issues at this stage are the most likely cause of variations in results.

#### 3.3.2. Cell-Based DCF Assay

To determine the formation of ROS in A549 cells, the DCF assay was performed [30]. Briefly, A549 cells were seeded into 96-well plates at a density of 2 × 10^4^ cells per well in a volume of 200 μL complete cell culture medium on the day prior to treatment. Cells were incubated for 60 min in 50 μM H_2_DCF-DA (Molecular Probes, Invitrogen, Basel, Switzerland) in HBSS at 37 °C and 5% CO_2_. After two washing steps with prewarmed HBSS the cells were treated with increasing concentrations of 100 μL Sin-1 and MWCNT, respectively, at 37 °C and 5% CO_2_. Fluorescence intensity was measured after 1, 2, 3 and 4 h from the same plate using a FLX800 fluorescence microplate reader (BioTEK Instruments, Winooski, VT, USA) at an excitation wavelength of 485 nm and an emission wavelength of 528 nm. Only values from the 3-hour reading point are shown in the Results Section.

#### 3.3.3. Cell-Free Interference Controls

##### 3.3.3.1. Intrinsic ROS Activity of NPs

To assess the intrinsic activity of MWCNTs to process the H_2_DCF molecule to its fluorescent form (DCF), the H_2_DCF-DA has to be deacetylated prior to incubation with increasing MWCNT concentrations. Therefore, 1 volume (e.g., 1 mL) of a 1 mM stock solution of H_2_DCF-DA in methanol was mixed with 20 volumes (e.g., 20 mL) of 0.01 M NaOH in a beaker protected from light and stirred for 30 min at room temperature. Adding 75 volumes (75 mL) of 33 mM NaH_2_PO_4_ stopped the reaction. The resulting solution contains 50 μM H_2_DCF.

In a 96-well plate, 50 μL of the 50 μM H_2_DCF were mixed with increasing concentrations of Sin-1 and MWCNTs, respectively, resulting in 100 μL total volume that was comparable to the cell-based assay. However, the concentrations are halved. Following the cell-based assay, fluorescence intensities were measured after 1, 2, 3 and 4 h of incubation. Only the 3-hour reading points are shown.

##### 3.3.3.2. Quenching Effect of NPs

The fluorescent dye DCF (Sigma, Buchs, Switzerland) is commercially available. In a 96-well plate, 50 μL of a 2.5 μM solution of DCF were mixed with 50 μL of increasing MWCNT concentrations and fluorescence intensities were measured as described above.

### 3.4. Assessment of Cytotoxicity Using the MTS Cell Viability Assay

CellTiter 96^®^ AQ_ueous_ One Solution (Promega, Duebendorf, Switzerland) was used according to the manufacturer’s protocol. Briefly, A549 cells were seeded into 96-well plates at a density of 8000 cells per well in 200 μL complete cell culture medium on the day prior to treatment. For each time point (3 h, 24 h) a separate plate is necessary. Cells were then incubated with increasing concentrations of Sin-1 or CdSO_4_ in 100 μL complete cell culture medium, respectively, at 37 °C and 5% CO_2_. After 3 and 24 h medium was replaced by 120 μL MTS working solution (composed of 100 μL phenol red free RPMI plus 20 μL MTS). After one hour of incubation at 37 °C and 5% CO_2_ absorbance was measured at 490 nm. Data are presented in % of the untreated control sample and represent the mean of 3 independent experiments and its standard deviation.

### 3.5. Data Processing (MTS & DCF)

Blank samples containing no cells (or in case of cell-free interference assessment no H_2_DCF) but treated exactly the same way (with NP or chemical control stimuli, all washing steps (if necessary), *etc.*) were always run in parallel on the same 96-well plate. Values given in the graphs are blank-corrected arbitrary units (a.u.).

## 4. Conclusions

The production of ROS plays a central role in cellular reactions involving NPs. To take this into account, any ROS-detecting assay needs to measure ROS production as reliably and reproducibly as possible. Furthermore, an internal measuring scale has to be established to enable traceability and finally comparability. Such an internal measuring stick is in most cases a chemical compound that functions as a positive control. A concentration series of the chemical reaction control allows the determination of its EC_50_ value, thereby proving correct functioning of the assay within very narrow quality limits.

Our results show that Sin-1 can be used as a qualitative control to induce H_2_DCF processing in a cellular, as well as a cell-free environment, only answering the one question: Did the assay work: Yes or No? However, because of the large variations in the observed fluorescence values between independent experiments, it is not possible to use Sin-1 as a quantitative internal measuring scale for the DCF assay. Furthermore, we think it is generally very hard to find a suitable chemical reaction control that functions as a reliable quantitative measuring scale for any *in vitro* ROS-detecting assay. Theoretically, the compound of interest has to fulfill the following requirements:

- be non-toxic at a radical generating concentration;
- be chemically stable without solvent;
- be dilutable in water (biological fluids);
- generate a stable rate of radical formation in solution/suspension over time (months/years).

Because of their high surface to volume ratio and reactive surface in general, certain NPs might fulfill (at least some of) these requirements. In addition, they have to fulfill the criteria of a reference material and should (at best), be generated in large amounts by a national measurement institute.

However, certain aspects still have to be kept in mind; for example, agglomeration of NPs in cell culture media and the associated different sedimentation/diffusion behaviors and, as a consequence, unknown dosage on the cells (effective dose). NP interference (quenching) constitutes an additional factor to be considered. Despite the obvious NP-related issues, we suggest searching for suitable NPs meeting these criteria that induce dose-dependent levels of ROS. These particles should then be introduced as a semiquantitative internal measuring scale. Calibration of this scale would then allow categorization of unknown chemicals, particles, any type of compound, as a “light”, “medium” or “severe” ROS-inducing agent.

## References

[1] Riehemann K., Schneider S.W., Luger T.A., Godin B., Ferrari M., Fuchs H. (2009). Nanomedicine-challenge and perspectives. Angew. Chem. Int. Ed. Engl.

[2] Nel A., Xia T., Madler L., Li N. (2006). Toxic potential of materials at the nanolevel. Science.

[3] Warheit D.B. (2008). How meaningful are the results of nanotoxicity studies in the absence of adequate material characterization?. Toxicol. Sci.

[4] Warheit D.B., Laurence B.R., Reed K.L., Roach D.H., Reynolds G.A., Webb T.R. (2004). Comparative pulmonary toxicity assessment of single-wall carbon nanotubes in rats. Toxicol. Sci.

[5] Warheit D.B., Webb T.R., Reed K.L., Frerichs S., Sayes C.M. (2007). Pulmonary toxicity study in rats with three forms of ultrafine-TiO2 particles: Differential responses related to surface properties. Toxicology.

[6] Bury N.R., Wood C.M. (1999). Mechanism of branchial apical silver uptake by rainbow trout is via the proton-coupled Na(+) channel. Am. J. Physiol.

[7] Fabrega J., Luoma S.N., Tyler C.R., Galloway T.S., Lead J.R. (2011). Silver nanoparticles: Behaviour and effects in the aquatic environment. Environ. Int.

[8] Aruoja V., Dubourguier H.C., Kasemets K., Kahru A. (2009). Toxicity of nanoparticles of CuO, ZnO and TiO_2_ to microalgae *Pseudokirchneriella subcapitata*. Sci. Total Environ.

[9] Ganesh R., Smeraldi J., Hosseini T., Khatib L., Olson B.H., Rosso D. (2010). Evaluation of nanocopper removal and toxicity in municipal wastewaters. Environ. Sci. Technol.

[10] Griffitt R.J., Luo J., Gao J., Bonzongo J.C., Barber D.S. (2008). Effects of particle composition and species on toxicity of metallic nanomaterials in aquatic organisms. Environ. Toxicol. Chem.

[11] Griffitt R.J., Weil R., Hyndman K.A., Denslow N.D., Powers K., Taylor D., Barber D.S. (2007). Exposure to copper nanoparticles causes gill injury and acute lethality in zebrafish (*Danio rerio*). Environ. Sci. Technol.

[12] Heng B.C., Zhao X., Tan E.C., Khamis N., Assodani A., Xiong S., Ruedl C., Ng K.W., Loo J.S. (2011). Evaluation of the cytotoxic and inflammatory potential of differentially shaped zinc oxide nanoparticles. Arch. Toxicol.

[13] Karlsson H.L., Cronholm P., Gustafsson J., Moller L. (2008). Copper oxide nanoparticles are highly toxic: A comparison between metal oxide nanoparticles and carbon nanotubes. Chem. Res. Toxicol.

[14] Kim J.S., Kuk E., Yu K.N., Kim J.H., Park S.J., Lee H.J., Kim S.H., Park Y.K., Park Y.H., Hwang C.Y. (2007). Antimicrobial effects of silver nanoparticles. Nanomedicine.

[15] Pujalte I., Passagne I., Brouillaud B., Treguer M., Durand E., Ohayon-Courtes C., L’Azou B. (2011). Cytotoxicity and oxidative stress induced by different metallic nanoparticles on human kidney cells. Part. Fibre Toxicol.

[16] Hancock J.T., Desikan R., Neill S.J. (2001). Role of reactive oxygen species in cell signalling pathways. Biochem. Soc. Trans.

[17] Donaldson K., Stone V., Borm P.J., Jimenez L.A., Gilmour P.S., Schins R.P., Knaapen A.M., Rahman I., Faux S.P., Brown D.M. (2003). Oxidative stress and calcium signaling in the adverse effects of environmental particles (PM10). Free Radic Biol. Med.

[18] Latella L., Sacco A., Pajalunga D., Tiainen M., Macera D., D’Angelo M., Felici A., Sacchi A., Crescenzi M. (2001). Reconstitution of cyclin D1-associated kinase activity drives terminally differentiated cells into the cell cycle. Mol. Cell Biol.

[19] Johnston H.J., Hutchison G.R., Christensen F.M., Peters S., Hankin S., Aschberger K., Stone V. (2010). A critical review of the biological mechanisms underlying the *in vivo* and *in vitro* toxicity of carbon nanotubes: The contribution of physico-chemical characteristics. Nanotoxicology.

[20] Donaldson K., Brown D.M., Mitchell C., Dineva M., Beswick P.H., Gilmour P., MacNee W. (1997). Free radical activity of PM10: Iron-mediated generation of hydroxyl radicals. Environ. Health Perspect.

[21] Held P. (2010). An Introduction to Reactive Oxygen Species.

[22] Ruch W., Cooper P.H., Baggiolini M. (1983). Assay of H_2_O_2_ production by macrophages and neutrophils with homovanillic acid and horse-radish peroxidase. J. Immunol. Methods.

[23] Zhou M., Diwu Z., Panchuk-Voloshina N., Haugland R.P. (1997). A stable nonfluorescent derivative of resorufin for the fluorometric determination of trace hydrogen peroxide: Applications in detecting the activity of phagocyte NADPH oxidase and other oxidases. Anal. Biochem.

[24] Halliwell B., Whiteman M. (2004). Measuring reactive species and oxidative damage *in vivo* and in cell culture: How should you do it and what do the results mean?. Br. J. Pharmacol.

[25] Gasser M., Wick P., Clift M.J., Blank F., Diener L., Yan B., Gehr P., Krug H.F., Rothen-Rutishauser B. (2012). Pulmonary surfactant coating of multi-walled carbon nanotubes (MWCNTs) influences their oxidative and pro-inflammatory potential *in vitro*. Part. Fibre Toxicol.

[26] Tarpey M.M., Wink D.A., Grisham M.B. (2004). Methods for detection of reactive metabolites of oxygen and nitrogen: *in vitro* and *in vivo* considerations. Am. J. Physiol. Regul. Integr. Comp. Physiol.

[27] Kalyanaraman B., Darley-Usmar V., Davies K.J., Dennery P.A., Forman H.J., Grisham M.B., Mann G.E., Moore K., Roberts L.J., Ischiropoulos H. (2012). Measuring reactive oxygen and nitrogen species with fluorescent probes: Challenges and limitations. Free Radic. Biol. Med.

[28] Rothe G., Valet G. (1990). Flow cytometric analysis of respiratory burst activity in phagocytes with hydroethidine and 2′,7′-dichlorofluorescin. J. Leukoc. Biol.

[29] Wardman P. (2008). Methods to measure the reactivity of peroxynitrite-derived oxidants toward reduced fluoresceins and rhodamines. Methods Enzymol.

[30] Shulaev V., Oliver D.J. (2006). Metabolic and proteomic markers for oxidative stress. New tools for reactive oxygen species research. Plant Physiol.

[31] Kohno M. (2010). Applications of electron spin resonance spectrometry for reactive oxygen species and reactive nitrogen species research. J. Clin. Biochem. Nutr.

[32] Hermans N., Cos P., Maes L., de Bruyne T., Vanden Berghe D., Vlietinck A.J., Pieters L. (2007). Challenges and pitfalls in antioxidant research. Curr. Med. Chem.

[33] Hartley D.P., Kolaja K.L., Reichard J., Petersen D.R. (1999). 4-Hydroxynonenal and malondialdehyde hepatic protein adducts in rats treated with carbon tetrachloride: Immunochemical detection and lobular localization. Toxicol. Appl. Pharmacol.

[34] van Acker S.A., van den Berg D.J., Tromp M.N., Griffioen D.H., van Bennekom W.P., van der Vijgh W.J., Bast A. (1996). Structural aspects of antioxidant activity of flavonoids. Free Radic. Biol. Med.

[35] Hodges D.M., DeLong J.M., Forney C.F., Prange R.K. (1999). Improving the thiobarbituric acid-reactive-substances assay for estimating lipid peroxidation in plant tissues containing anthocyanin and other interfering compounds. Planta.

[36] Byrdwell W.C., Neff W.E. (2002). Dual parallel electrospray ionization and atmospheric pressure chemical ionization mass spectrometry (MS), MS/MS and MS/MS/MS for the analysis of triacylglycerols and triacylglycerol oxidation products. Rapid Commun. Mass Spectrom.

[37] Buerki-Thurnherr T., Xiao L., Diener L., Arslan O., Hirsch C., Maeder-Althaus X., Grieder K., Wampfler B., Mathur S., Wick P. (2013). *In vitro* mechanistic study towards a better understanding of ZnO nanoparticle toxicity. Nanotoxicology.

[38] Keston A.S., Brandt R. (1965). The fluorometric analysis of ultramicro quantities of hydrogen peroxide. Anal. Biochem.

[39] Bureau International des Poids et Mesures (BIPM) (2012). International Vocabulary of Metrology: Basic and General Concepts and Associated Terms (VIPM). The Joint Committee for Guides in Metrology (JCGM).

[40] Hirsch C., Roesslein M., Krug H.F., Wick P. (2011). Nanomaterial cell interactions: Are current *in vitro* tests reliable?. Nanomedicine.

[41] Limbach L.K., Wick P., Manser P., Grass R.N., Bruinink A., Stark W.J. (2007). Exposure of engineered nanoparticles to human lung epithelial cells: Influence of chemical composition and catalytic activity on oxidative stress. Environ. Sci. Technol.

[42] Lipton S.A., Choi Y.B., Pan Z.H., Lei S.Z., Chen H.S., Sucher N.J., Loscalzo J., Singel D.J., Stamler J.S. (1993). A redox-based mechanism for the neuroprotective and neurodestructive effects of nitric oxide and related nitroso-compounds. Nature.

[43] Piret J.P., Jacques D., Audinot J.N., Mejia J., Boilan E., Noel F., Fransolet M., Demazy C., Lucas S., Saout C. (2012). Copper(II) oxide nanoparticles penetrate into HepG2 cells, exert cytotoxicity via oxidative stress and induce pro-inflammatory response. Nanoscale.

[44] Wang H., Joseph J.A. (1999). Quantifying cellular oxidative stress by dichlorofluorescein assay using microplate reader. Free Radic. Biol. Med.

[45] Ellison S.L.R., King B., Rösslein M., Salit M., Williams A., Eurachem CITAC Working Group (2003). Traceability in Chemical Measurement. A Guide to Achieving Comparable Results in Chemical Measurement.

[46] Lieber M., Smith B., Szakal A., Nelson-Rees W., Todaro G. (1976). A continuous tumor-cell line from a human lung carcinoma with properties of type II alveolar epithelial cells. Int. J. Cancer.

[47] Thurnherr T., Su D., Diener L., Weinberg G., Masnser P., Pfänder N., Arrigo R., Schuster M.E., Wick P., Krug H.F. (2009). Comprehensive evaluation of *in vitro* toxicity of three large-scale produced carbon nanotubes on human Jurkat T cells ans a comparison to crocidolite asbestos. Nanotoxicology.

[48] Voelkel K., Krug H.F., Diabate S. (2003). Formation of reactive oxygen species in rat epithelial cells upon stimulation with fly ash. J. Biosci.

